# [Corrigendum] Identification of key pathways and candidate genes in pancreatic ductal adenocarcinoma using bioinformatics analysis

**DOI:** 10.3892/ol.2026.15920

**Published:** 2026-10-26

**Authors:** Yiping He, Yan Liu, Jianping Gong, Changan Liu, Hua Zhang, Hao Wu

Oncol Lett 17: 3751-3764, 2019; DOI: 10.3892/ol.2019.10041

Subsequently to the publication of the above paper, an interested reader drew to the authors' attention that they had noticed potential errors/discrepancies with the descriptions of the data as reported in Table I and Fig. 1. Specifically, in Table I, the authors had stated: “A total of 424 DEGs were identified from the GSE28735 dataset, including 159 upregulated genes and 265 downregulated genes in PDAC tissues, compared to adjacent non-tumor tissue samples.” However, the reader considered that genes such as *LAMC2*, *GALNT5*, *TSPAN1*, *CTSE* and *POSTN*, which were labeled as “Downregulated” genes in Table I have generally been reported to be significantly upregulated in PDAC, and requested that the authors check this.

In response to the question raised, the authors re-analyzed their original data, and found that a clerical error had been made in terms of the data annotation. Concerning Table I, in the body of the table, the genes listed as “Up-downregulated” should have been labeled as “Down-regulated” genes, and *vice versa;* in addition, the designation of up- and down-regulated genes in the Table legend also needed to be changed. A corrected version of Table I, showing changes highlighted in bold, is shown on the next page.

Furthermore, the following changes should be noted for the text of the article:

- In the Results section on p. 3752, the second and third sentences of the first paragraph should have read as follows: “A total of 424 DEGs were identified from GSE28735, including 1**59 downregulated and 265 upregulated** genes (Table I). The heat map of DEG expression, presenting the **top 50 downregulated and 50 upregulated** genes was constructed using the web-based tool Morpheus (Fig. 1).
- The first sentence of the *PPI and modular analysis in pancreatic cancer* subsection of the Results on the same page should have read as follows: “Using the STRING online database and Cytoscape software analysis, a total of 386 DEGs (**143 downregulated and 243 upregulated** genes) of the 424 commonly altered DEGs...”.
- The final two sentences of this subsection should have read as follows: “Among the **downregulated** DEGs, a total of 143 DEGs were filtered into the DEG PPI network complex including 143 nodes and 263 edges (Fig. 2B), which were mainly associated with ‘digestion’, ‘serine-type peptidase activity’ and the ‘extracellular region’ (Table V). Among the **upregulated** DEGs, a total of 143 DEGs were filtered into the DEGs PPI network complex including 243 nodes and 497 edges (Fig. 2C), which were mainly associated with ‘ECM organization’, ‘ECM structural constituents’ and the ‘extracellular region’ (Table VI).”
- On p. 3756, left-hand column, the sentences at the beginning of the new paragraph of the Discussion should have read as follows: “Furthermore, KEGG pathway analysis indicated that the **downregulated** DEGs were involved in pancreatic secretion pathways and protein and lipid digestion and absorption pathways. Existing studies revealed that metabolic change is considered one of the characteristics of cancer, especially the dysfunction of pancreatic secretion (22,11). In pancreatic cancer, metabolic changes are prominent in protein and lipid digestion and absorption pathways (23). The **up-regulated** DEGs were associated with ‘ECM-receptor interaction’, ‘focal adhesion’ and the ‘PI3K-Akt signaling’ pathways.”
- On p. 3761, left-hand column, the sentence at the start of the sixth paragraph of the Discussion should have read as follows: “For the **downregulated** DEGs, module analysis of the PPI network revealed that they were associated with...”.
- Also on p. 3761, the right-hand column, the sentence at the start of the seventh paragraph of the Discussion should have read as follows: “The **upregulated** DEGs were associated with ECM-receptor interactions, focal adhesion and the PI3K-Akt signaling pathway (46).”
- The title and legend for Fig. 2 should have read as follows: “Figure 2. DEGs PPI network complex in PDAC. (A) All 386 DEG interaction networks. **(B) The downregulated DEG interaction network. (C) The upregulated DEG interaction network.**”
- Finally, the titles of Tables II, III, V and VI should have read as follows:

Table II. GO analysis of **downregulated** DEGs associated with PDAC.

Table III. GO analysis of **upregulated** DEGs associated with PDAC.

Table V. The enriched pathways of **downregulated DEGs** PPI network in PDAC.

Table VI. The enriched pathways of **upregulated DEGs** PPI network in PDAC.

The authors regret the clerical errors that were made during the writing of this paper, and express their thanks to the reader who drew this matter to their attention. They are also grateful to the editor of *Oncology Letters* for allowing them the opportunity to publish this Corrigendum. All the authors agree with the publication of this corrigendum; furthermore, they apologize to the readership for any inconvenience caused.

